# Supplementary material for: COZOID: contact zone identifier for visual analysis of protein-protein interactions
Source: BMC Bioinformatics. 2018 Apr 6;19:125. doi: 10.1186/s12859-018-2113-6 (PMC5889581; doi:10.1186/s12859-018-2113-6)
Supplement: Supplementary file 2 — Software build. Executable binary file of the software tool. (ZIP 118,784 kb) [file 12859_2018_2113_MOESM2_ESM.zip › cozoid/java/docs/beansbinding-1.2.1-doc.zip!/org/jdesktop/swingbinding/package-use.html]

Uses of Package org.jdesktop.swingbinding (Beans Binding)


---


|  |  |  |
| --- | --- | --- |
| **Overview** \| **Package** \| Class \| **Use** \| **Tree** \| **Deprecated** \| **Index** \| **Help** | |  |
| PREV   NEXT | **FRAMES**    **NO FRAMES**     **All Classes** |  |


---


## **Uses of Package org.jdesktop.swingbinding**

| Packages that use org.jdesktop.swingbinding | |
| --- | --- |
| **org.jdesktop.swingbinding** | Provides support for binding to complex Swing components, and documentation on the interesting Swing properties to bind to. |

Classes in org.jdesktop.swingbinding used by org.jdesktop.swingbinding

****JComboBoxBinding****
  
          Binds a `List` of objects to act as the items of a `JComboBox`.

****JListBinding****
  
          Binds a `List` of objects to act as the elements of a `JList`.

****JListBinding.DetailBinding****
  
          `DetailBinding` represents a binding between a property of the elements
in the `JListBinding's` source `List`, and the values shown in
the `JList`.

****JTableBinding****
  
          Binds a `List` of objects to act as the rows of a `JTable`.

****JTableBinding.ColumnBinding****
  
          `ColumnBinding` represents a binding between a property of the elements
in the `JTableBinding's` source `List`, and a column in the table.

---


|  |  |  |
| --- | --- | --- |
| **Overview** \| **Package** \| Class \| **Use** \| **Tree** \| **Deprecated** \| **Index** \| **Help** | |  |
| PREV   NEXT | **FRAMES**    **NO FRAMES**     **All Classes** |  |


---
